# Community Participation in Chagas Disease Vector Surveillance: Systematic Review

**DOI:** 10.1371/journal.pntd.0001207

**Published:** 2011-06-21

**Authors:** Fernando Abad-Franch, M. Celeste Vega, Miriam S. Rolón, Walter S. Santos, Antonieta Rojas de Arias

**Affiliations:** 1 Instituto Leônidas e Maria Deane – Fiocruz Amazônia, Manaus, Brazil; 2 Centro para el Desarrollo de la Investigación Científica, Asunción, Paraguay; 3 Pan American Health Organization, Asunción, Paraguay; Universidad de Buenos Aires, Argentina

## Abstract

**Background:**

Vector control has substantially reduced Chagas disease (ChD) incidence. However, transmission by household-reinfesting triatomines persists, suggesting that entomological surveillance should play a crucial role in the long-term interruption of transmission. Yet, infestation foci become smaller and harder to detect as vector control proceeds, and highly sensitive surveillance methods are needed. Community participation (CP) and vector-detection devices (VDDs) are both thought to enhance surveillance, but this remains to be thoroughly assessed.

**Methodology/Principal Findings:**

We searched Medline, Web of Knowledge, Scopus, LILACS, SciELO, the bibliographies of retrieved studies, and our own records. Data from studies describing vector control and/or surveillance interventions were extracted by two reviewers. Outcomes of primary interest included changes in infestation rates and the detection of infestation/reinfestation foci. Most results likely depended on study- and site-specific conditions, precluding meta-analysis, but we re-analysed data from studies comparing vector control and detection methods whenever possible. Results confirm that professional, insecticide-based vector control is highly effective, but also show that reinfestation by native triatomines is common and widespread across Latin America. Bug notification by householders (the simplest CP-based strategy) significantly boosts vector detection probabilities; in comparison, both active searches and VDDs perform poorly, although they might in some cases complement each other.

**Conclusions/Significance:**

CP should become a strategic component of ChD surveillance, but only professional insecticide spraying seems consistently effective at eliminating infestation foci. Involvement of stakeholders at all process stages, from planning to evaluation, would probably enhance such CP-based strategies.

## Author Summary

Blood-sucking triatomine bugs are the vectors of Chagas disease, a potentially fatal illness that affects millions in Latin America. With no vaccines available, prevention heavily depends on controlling household-infesting triatomines. Insecticide-spraying campaigns have effectively reduced incidence, but persistent household reinfestation can result in disease re-emergence. What, then, is the best strategy to keep houses free of triatomines and thus interrupt disease transmission in the long run? We reviewed published evidence to (i) assess the effectiveness of insecticide-based vector control, gauging the importance of reinfestation; (ii) compare the efficacy of programme-based (with households periodically visited by trained staff) and community-based (with residents reporting suspect vectors found in their homes) surveillance strategies; and (iii) evaluate the performance of alternative vector-detection methods. The results confirm that insecticide-based vector control is highly effective, but also that persistent house reinfestation is a general trend across Latin America. Surveillance systems are significantly more effective when householders report suspect bugs than when programme staff search houses, either manually or using vector-detection devices. Our results clearly support the view that long-term vector surveillance will be necessary for sustained Chagas disease control – and that community participation can substantially contribute to this aim.

## Introduction

Chagas disease still imposes a heavy burden on most Latin American countries, with about 10–12 million people infected by *Trypanosoma cruzi* [1], [2]. Multinational control initiatives have since the early 1990s drastically reduced prevalence and incidence, mainly through insecticide-based elimination of domestic vector populations (blood-sucking bugs of the subfamily Triatominae) [3] and systematic screening of blood donors with highly sensitive serological tests [1], [2], [4], [5]. In spite of these advances, vector-borne transmission is estimated to cause about 40,000 new infections per year [6]. Reinfestation of treated households by native vectors as the residual effect of insecticides vanishes is the most likely mechanism underlying such persistent transmission [7]. Similarly, outbreaks of acute Chagas disease have been attributed to the contamination of foodstuffs by infected adult (i.e., winged) triatomines that invade premises where food is processed or stored [8]–[12]. In Amazonia and other humid forest ecoregions, where the bugs rarely colonise inside houses, endemic, low-intensity transmission seems also mediated by adventitious, household-invading triatomines [13]–[15]. In addition, there is growing concern that insecticide-resistant vector populations, such as those detected in southern South America [16], [17], may threaten effective disease prevention.

This rapid overview shows why sustained Chagas disease control is believed to require some sort of longitudinal, long-term surveillance system capable of detecting and eliminating household infestation foci [1], [18]. Surveillance typically relies on the periodical inspection of households by trained personnel. Active vector searches are performed with or without the aid of chemical ‘flush-out’ agents such as low-dose pyrethroid dilutions, and infestation foci are eliminated by insecticide spraying when discovered [18].

However, detecting the vectors can be difficult, particularly when only small populations occur within or around households. In fact, vector colonies are expected to become rarer and smaller as control programmes proceed, and managers are progressively less prone to fund costly active surveillance resulting in few detection events. A number of vector-detection devices have been designed in an attempt to enhance surveillance; most consist of boxes that triatomines can use as refuges or of paper sheets or calendars where the typical faecal streaks of the bugs can be identified [19]–[27]. Such ‘sensing devices’ are placed within households or in annex structures and checked periodically for bugs or their traces, supposedly reducing the costs of surveillance while retaining adequate sensitivity [26]–[29].

Finally, and since the early vector control trials, there has been a perception that resident householders may have better chances of discovering bugs in their own homes than a visiting team searching the house for a few minutes every several months [30]–[32]. ‘Community participation’ in entomological surveillance gained extra momentum with the Declaration of Alma Ata [33], [34], which “…encouraged approaches to health care that incorporated community participation and community development” (ref. [34], p. 1). Experiences involving community participation in Chagas disease control have been described in several settings across Latin America [18], [30], [31]; they seem to converge towards an encouraging overall picture, and the Chagas disease example has accordingly been praised in several subjective reviews (e.g., [35], [36]).

However, the effectiveness of these diverse strategies for Chagas disease vector surveillance, including community participation, has not been thoroughly and objectively assessed at the continental scale. With the aim of filling this gap, we systematically reviewed the published evidence on this issue, tackling specifically the following major questions: (i) How common and important is the phenomenon of house reinfestation by triatomine bugs after control interventions?; (ii) How effective are different vector surveillance strategies at detecting infestation/reinfestation foci?; (iii) To what extent have community participation and empowerment been effectively promoted?; and, finally, (iv) Can available strategic options be condensed in overarching recommendations for surveillance that apply across the highly diverse ecological and social-cultural settings where the problem is present?

## Methods

The review protocol is available upon request from the corresponding author. This review was carried out in the context of a collaborative project led by the Inter-American Development Bank, and was not formally registered. We searched Medline, ISI Web of Knowledge, Scopus, LILACS, and SciELO; the major query argument was “Triatomin* AND (Control OR Surveillance)”. Searches retrieved records from 1948 to 2009, including additional documents identified by searching bibliographies and in the authors' records. This search strategy aimed at recovering documents describing vector control interventions, with or without surveillance, so that post-control reinfestation trends could also be assessed. Only documents describing field interventions aimed at the control and/or surveillance of domestic Chagas disease vectors were included in the full review process. Descriptive (non-intervention) reports, results of research with laboratory or experimental vector populations, expert reviews, and opinion or commentary pieces were either excluded or used only for the introduction and/or discussion.

**Figure 1:**
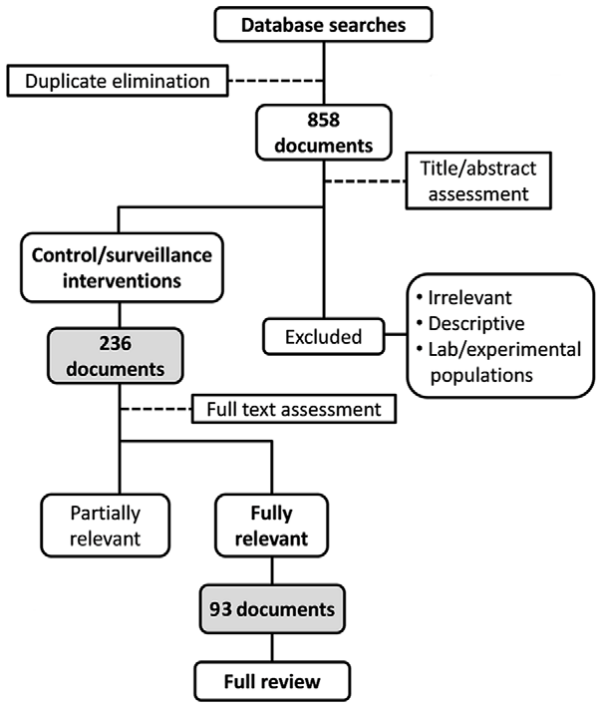
Flow diagram of the systematic review process.

We were particularly interested in comparing strategies involving institutional (by professional staff) or participatory surveillance. We also compared alternative methods for vector detection, including active searches, vector-detection devices, and community participation. Major outcomes included household infestation/reinfestation indices (or, in some cases, bug catches) and vector detection rates. Inclusion/exclusion of documents was assessed independently by ARdA and FA-F, and discrepancies resolved by consensus. Figure 1 presents the flow diagram of the review process. Data were independently extracted by ARdA and FA-F using predefined data fields inspired by the *Guide to Community Preventive Services* [37] (www.thecommunityguide.org) and including study quality indicators. FA-F revised data extraction results and resolved inconsistencies by re-checking the original documents. The following items were considered: (1) study classification (study design, intervention components, whether or not the intervention was part of a broader initiative, outcomes); (2) descriptive information, including (2.i) description of the intervention (what was done, how, where and by whom it was done, theoretical basis of the intervention, types of organisation involved, whether or not there was any intervention in a control group), (2.ii) study characteristics (place, time, population, settings, outcome measurement, whether or not there was a measurement of exposure to the intervention), (2.iii) results (primary results, sample and effect sizes), and (2.iv) applicability in settings other than the actual study one (direct and indirect costs, harms and benefits, implementation process, and whether the community participated at each stage of the process – design, pre-implementation, effecting, and evaluation); and (3) study quality, including quality of descriptions, sampling (universe, eligibility and selection of participants, sample size, potential sampling biases), effect measurements, data analyses (statistics, confounders, repeated measures or other sources of non-independence), and interpretation of results (rate of adherence, control and assessment of potential confounders and sources of bias). Relevant references and other details deemed important were also recorded. The protocol required extracting detailed demographic data about intervention and control or indirectly affected populations. Such information was however absent from or incomplete in most studies; this, together with the fact that the outcomes of primary interest refer to households, not individual people, led us to exclude these items from the protocol during the course of the review.

The often important morphological, ecological and behavioural differences among triatomine bug species [3], combined with the likely sensitivity of results to study-specific (methods, research team performance) and site-specific conditions (vector density, household building materials and structure), led us to avoid estimating meta-analytical summary effects from different reports. Inadequate design and/or reporting of several studies were further factors hindering meta-analysis. When enough information was given in the original reports, we nonetheless re-analysed data from studies comparing control strategies (in terms of household infestation rates) and vector detection techniques (in terms of detection rates). Whenever possible, we used McNemar's tests for correlated proportions [38], with odds ratios (OR) estimated as the ratio of discordant results. When independence of observations was likely, or in the absence of complete data on repeated observations, ORs were estimated from standard contingency tables [39]. Approximate OR 95% confidence intervals (95%CI) were calculated by assuming normality of log-odds [39]. The VassarStats online facility (http://faculty.vassar.edu/lowry/VassarStats.html) and Microsoft Office Excel® spreadsheets were used for the analyses.

## Results

### Overall results

Database searches retrieved 1,342 candidate documents; elimination of duplicates yielded 858 unique records (Figure 1) in English, Spanish, or Portuguese. Assessment of titles and abstracts yielded five groups: (a) documents apparently describing control and/or surveillance interventions (236 records), (b) non-intervention studies, (c) studies with laboratory or experimental vector populations, (d) subjective reviews and opinion pieces, and (e) reports clearly irrelevant to our review. Evaluation of group (a) documents against inclusion criteria identified 93 reports for full data extraction [Supporting Information, List S1]; of the remaining 143 (plus several additional references), 26 studies [Supporting Information, List S2] were also used for partial quantitative assessments, and the rest were considered as supplementary sources of qualitative information for the introduction and/or discussion.

**Figure 2:**
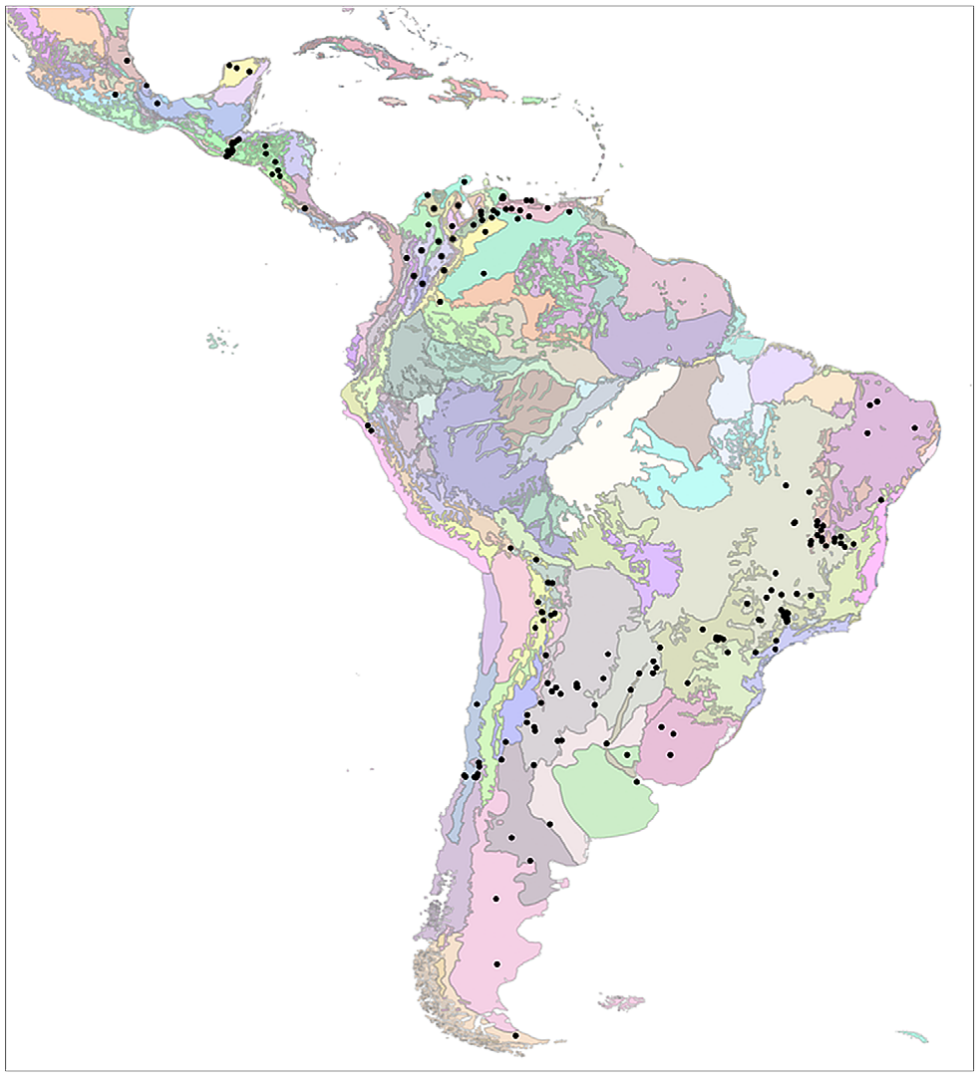
Geographical-ecological coverage of studies on Chagas disease vector control and surveillance. Study site locations (black dots) are overlaid on the World Wildlife Fund ecoregional map of Latin America (available with detailed ecoregion legends at www.conserveonline.org/docs/2001/06lac_ecoregions.jpg).

The spatial and ecological coverage of our review is represented in Figure 2. Only 11 randomised trials [40]–[50] were identified, with just one crudely assessing a community-based intervention [50] and four describing different aspects of the same trial [44]–[47]. Over half of the studies dealt directly or indirectly with different strategies for household-level vector surveillance. Interventions ranged from insecticide spraying (the most frequent) to educational activities, with a few studies describing alternative control approaches such as environmental management [51]–[58] or insecticide-treated materials [48], [49], [59]. Most studies measured intervention effects as reductions in household infestation rates (through entomological surveys) or as vector detection rates (through detection records). While the quality of the descriptions was generally adequate, analytical procedures were often dubious; for instance, albeit many studies describe results in which the same sampling units were assessed more than once (e.g., before-after, time-series) or by more than one method (e.g., vector-detection studies), only a few apply statistical tests suited for repeated measures or other sources of non-independence of observations.

Collaborative efforts involving both academic institutions and official public health agencies were common (∼70% of studies), a typical historical trait of Chagas disease vector control [60]. Even though sustainability was discussed in several documents, detailed assessment of the costs (monetary and not) and potential unintended benefits and harms was rare. Forty-eight reports described some sort of ‘community participation’ in the intervention; however, none of them explicitly stated that participation took place at the design stage, and only three describe a participatory evaluation process [47], [58], [61]. In contrast, local residents helped carry out the intervention in 45 studies, mainly by reporting vectors caught in their homes; in 20, the community was also involved in the pre-implementation phase.

### Control effectiveness and the role of surveillance

Since Carlos Chagas historic paper [62], vector control has become the cornerstone of primary Chagas disease prevention [60], [63]. Pioneering attempts involved chemical (including cyanide gas) and physical means (including flamethrowers) [64]. The failure of DDT in controlling triatomines was followed by substantial optimism when HCH (lindane) proved successful in early trials in Brazil [65], [66], Argentina [67], and Chile [68]. The effectiveness of insecticide-based control kept improving as new chemicals and better formulations, with longer residual effects and lower toxicity, were introduced [40]–[42], [45], [69], [70]. Synthetic pyrethroids are now widely used and continue to be very efficient [71]–[75]; yet, recent research suggests that resistance may be widespread among some *Triatoma infestans* populations [16], [17], and insecticides are less effective in peridomestic environments [43], [76]. The top-quality report (in terms of sample size, design, and data treatment) we retrieved shows that peridomestic *T. infestans* foci reappear quickly after spraying (albeit with lower-density colonies) and that standard deltamethrin application with manual sprayers performs better than more sophisticated techniques [43].

Table 1 summarises the results of major reports on Chagas disease vector control [5],[18],[44],[57],[61],[63],[71]–[73],[77]–[113]. Overall, these studies unequivocally show that household insecticide spraying has successfully reduced infestation rates throughout Latin America, but also that reinfestation of dwellings by native vector species is common, spatially widespread, and temporally persistent. In many cases, the elimination of introduced populations was closely followed by the occupation of vacant niches by ‘secondary’ vector species, suggesting that the former had displaced the latter upon introduction [114], [115].

**Table 1:** Chagas disease vector control interventions: effectiveness, reinfestation trends, and the replacement of introduced species by native vectors.

Ref.	Comparison	Intervention	Vectors	Setting	Units, size	Main results, comments and caveats
[77]	Before-after	HCH	Ti*, Pm	Brazil (Cerrado, Bahia Interior Forests)	Infestation rates, 324 DUs	Infestation odds ∼6 (3 to 12) times lower after treatment; treated DUs near non-treated localities were ∼5 times less protected. (Analyses assume observations in each DU and time-point are independent)
[78]	Time-series (1951–64)	HCH yr 6–8	Ti*, Pm	Brazil (Cerrado, Bahia Interior Forests)	Capture events by control agents and bugs captured by control agents	Median annual capture events before-during-after treatment: Ti, 95-13-24; Pm, 31-14-36.5. Median number of bugs captured before-during-after treatment: Ti, 4,405-1,802-274; Pm, 138-72-186. (No information on number of DUs studied each year)
[79]	Time-series (1950–54; 1960–69)	HCH	Ti*, Pm	Brazil (Alto Paraná Atlantic Forest, Cerrado)	Number of bugs captured in DUs	Median annual capture (range) 1950–54: Ti, 1,330 (167–1,850); Pm, 14 (0–775). Median annual capture (range) 1960–69: Ti, 27 (3–440); Pm, 1,506 (678–3,741). (No information on number of DUs studied each year)
[80]	Before-after	HCH	Ti*, Ts, Pm	Brazil (Cerrado)	Vector presence, ∼500 localities	Ti virtually disappears; Ts persists in DUs of ∼45% of localities; Pm presence rises from 26% to 41% of localities
[63]	Time-series (1983; 1986–99)	Mainly Deltamethrin	Ti*, several native spp.	Brazil (several ecoregions across the country)	Number of bugs captured by control agents per yr countrywide	Ti falls from >84,000 (in 1983) to <600 (99.3% reduction); native spp. fall from ∼540,000 to ∼252,000 (53.2% reduction). (No information on number of DUs studied each year)
[81]	Time-series (1977–78, six visits) with control	HCH mo 1	Ti*, Pm	Brazil (Cerrado)	Infestation rates, 133 houses+496 annexes (control area: 39+101)	Before: house infestation by Ti ∼6.5 higher than by Pm; annex infestation by Pm ∼4.5 higher than by Ti. After: Ti disappears; Pm annex infestation rises from 1% to 3.4%, and the species reappears in houses. Control area: Ti stable in houses, reduction in annexes; Pm stable. (Probable non-independence of some observations)
[82]	Time-series (1976–77, six visits) with control	HCH mo 1	Ti*, Ts	Brazil (Cerrado)	Infestation rates, 301 houses+726 annexes (control area: 61+174)	Overall DU infestation falls from 35–40% to 1–3%; annex infestation recovers and, for Ts, increases from ∼1% to ∼7%. Ti was not eliminated. Ts increased more sharply in treatment than control areas. (Probable non-independence of some observations)
[83]–[87]	Time-series (1979–91+1999)	HCH yr 2; Deltamethrin, surveillance from yr 5	Ti*, Ts	Brazil (Cerrado)	Infestation rates, median 535 DUs/year (range 237–1,486)	Pre-intervention: 62% DUs infested (Ti 61%; Ts 0.5%); HCH: mean DU infestation 31.6% (Ti 21.8%; Ts 8.2%); Deltamethrin: mean 28.3% (Ti 6.3%, disappears in 1991; Ts 17.1%, rises from 10.7% to 37.7%); re-assessment 1999: overall DU infestation 8.75%. Assuming independent observations: OR (1991 vs. 1979) 0.23, 95%CI 0.17–0.32; OR (1999 vs. 1991) 0.38, 95%CI 0.18–0.35
[86], [87]	Time-series (1984–86+1999)	Deltamethrin, surveillance from 1985	Ti*, Ts	Brazil (Cerrado)	Infestation rates, ∼210 DUs/year (1,140 in 1999)	Infestation falls from 56.3% to 3.9% in 1986; OR 0.03, 95%CI 0.02–0.07. Ti not found in the 1999 re-assessment, when 12% of DUs (in 74% of localities) were infested, mainly by peridomestic Ts; OR 3.4, 95% CI 1.6–7. (OR calculations assume independence of observations)
[63], [73], [88]	Time-series (1953, 58, 63, 68, 73–2008)	HCH 1963; focal Deltamethrin 1973–84; surveillance from 1984	Ti*, Ts, Pm	Brazil (mainly Cerrado and Alto Paraná Atlantic Forests)	Number of bugs captured by control agents	Ti decreases from >60,000 in 1953 to 0 in 2000, with the last focus (131 bugs) eliminated in 1999; Ts reaches a maximum of >114,000 in 1968, then steadily decreases to ∼7,000 bugs/yr from 1990 on; Pm reaches a maximum of >10,500 in 1968, then decreases to ∼2,800 bugs/yr in the 80s, ∼1,100 in the 90s, and ∼500 bugs/yr in 2000–2008. The number of DUs searched each year varied markedly: >600,000/yr up to 1973, ∼450,000/yr 1974–84, ∼20,000/yr 1985–88, and <50,000/yr from 1989
[63]	Time-series (1973–97)	Focal Deltamethrin yr 1–12; surveillance yr 13–25	Ti*, Ts, Pm	Brazil (mainly Cerrado and Alto Paraná Atlantic Forests)	Species-pooled infestation rates in domiciles and peridomiciles	Domiciles: initial slight decrease (0.7 to 0.1%); increase after 1990, up to 2.2% (second-order polynomial fit: y = 0.01x^2^−0.19x+0.97, R^2^ = 0.94). Peridomiciles: initially stable (∼1.4%); increase to ∼10% after 1989 (second-order polynomial fit: y = 0.04x^2^−0.65x+3.01, R^2^ = 0.9). Increases attributed to participatory surveillance started by the late 80s. (No information on number of DUs studied each year)
[73]	Time-series (1968–2001)	Selective HCH; focal Deltamethrin 1973–84; surveillance from 1984	Ti*, Ts, Pm	Brazil (mainly Cerrado and Alto Paraná Atlantic Forests)	Species-pooled infestation rates in domiciles and peridomiciles	Domiciles: decline from ∼19% infested in 1968 to ∼6% (1973) and ∼1% from 1976 on. Peridomiciles: infestation falls from >35% up to 1971 to 25% in 1972, ∼15% in 1973–76, 5–10% in 1974–84, and <2.5% on average thereafter. (Approximate values taken from Figure 2 in the reference). (Note the discrepancy in peridomestic infestation rates with the data from ref. 63 above [∼10% after 1989])
[89]	Time-series (1993–99)	Not specified (probably pyrethroids)	Mainly Tb	Brazil (Caatinga, Cerrado)	“Capture index” (No. bugs/1,000 DUs searched)	Over 1 million DUs searched/yr; Tb dominant in areas where Ti was formerly the main vector. “Capture indices” higher in states within the Caatinga ecoregion (median 8.3, range 0–126). 19 additional native species, plus some residual Ti foci, were found in DUs
[90]	Before plus 2 follow-up assessments	Deltamethrin	Ts	Brazil (Cerrado, Caatinga, Campos Rupestres Montane Savannas)	Number of bugs captured, pre-treatment DU infestation rates	Pre-treatment: 772 Ts captured in 142 DUs (35%) and 192 peridomestic ecotopes (27.6%); first follow-up (after 7 mo): 405 Ts captured; second follow-up (1 yr post-treatment): 611 Ts captured. (No information on infestation rates or number of DUs and ecotopes studied in follow-up)
[91]	Before plus 3 follow-up assessments	Deltamethrin	Tb, Tps	Brazil (Caatinga)	Infestation rates, 277 DUs	Pre-treatment: 155 DUs (56%) infested; follow-up (4-mo intervals): 13.4%, 17.3%, and 31.8%. (Data not always congruent; for instance, reported pre-treatment rate [40.8%] and number of infested DUs [155])
[92]	Time-series (1977–90)	Not specified (pyrethroids after 1983)	Trv, Ti*	Brazil (Uruguayan Savanna)	DU infestation rates in 2 municipalities	Ti dominant until the mid-80s, only residual foci after 1986; Trv occasionally found in DUs before the 80s, replaces Ti after 1986. (National campaign to eradicate Ti began in 1983. No information on number of DUs studied each year)
[93]	Before-after	Deltamethrin	Ti	Argentina (Dry Chaco)	Infestation rates and number of bugs, 118 DUs	Pre-treatment: infestation 79.7%, 1,351 bugs captured; 3 yr after treatment: infestation 10.9%, 95 bugs captured. Infestation OR 0.03, 95%CI 0.02–0.07 (assuming independence) (Community-based surveillance was implemented)
[94]	Before plus 6 follow-up assessments	Deltamethrin	Ti	Argentina (Dry Chaco)	Infestation rates, ∼40 DUs on average	Pre-treatment: infestation 88%; 6 mo after: 0%; 1 yr after: 5%; 2 yr after: 24.4%; 3 yr after: 70%; 4 yr after: 94.4%; 5 yr after: 95.7%. Assuming independence: OR (yr 3 vs. yr 0) 0.25, 95%CI 0.07–0.9; OR (yr 5 vs. yr 3) 9.4. 95%CI 1.96–45.3
[95]	Follow-up after treatment	Deltamethrin	Ti	Argentina (Dry Chaco)	Infestation rates, 1,579 domestic and peridomestic ecotopes	Initial ∼2%; 6-mo interval assessments: average ∼1% for first three assessments, then increases to ∼3%, ∼7%, ∼9%, ∼8%, and reaches >30% in the last assessment. (Approximate values taken from Figure 1A in the reference)
[96]	Before-after	Deltamethrin	Ti	Argentina (Dry Chaco)	Infestation rates, 533 DUs initially; 89 localities	Pre-treatment infestation rate: 48.2%; 1 yr later, 383 DUs searched (only peridomicile) and 108 found infested (28.2%). Infestation of localities: 53% before, 39% after (McNemar OR 0.5, 95%CI 0.3–1.02)
[18]	Time-series (1984–2006)	Mainly Deltamethrin, fumigant canisters	Ti	Argentina (Dry Chaco)	Infestation rates, ∼300 DUs	Pre-treatment infestation 88%; 6 mo after 0%; recovery to pre-treatment levels in 5–7 yr; new interventions (community-based surveillance and selective control) reduce infestation to <5% for 4 yr, but it reaches >25% 4 yr later; thereafter, and for 7 more yr, infestation remains ∼10% on average
[5], [97]	Time-series (1980–2000)	National control programme	Mainly Ti (partly*)	Argentina (all ecoregions north of parallel 46S)	Overall DU infestation rates	∼30% infested DUs in 1980; >6% in 1992; <2% in 1999–2000. About 675,000 DUs sprayed in 1993–98, with >100,000 DUs/yr between 1991 and 2000; >820,000 DUs were under surveillance by 2000
[97]	Time-series (1964–2000)	National control programme	Mainly Ti (partly*)	Argentina (all ecoregions north of parallel 46S)	Province-specific DU infestation rate classes	The percentage of provinces with infestation rates >20% fell from 68.2% in 1964 to 60% (1982), 58.3% (1987), 22.2% (1992), and 5.5% (2000); for provinces with rates below 20%, the figures were 31.8%, 40%, 41.7%, 77.8%, and 94.4% for the same years. (*N* = 22, 15, 12, 18, and 18 provinces surveyed in each evaluation year)
[57], [72]	Before-after and follow-up at 6-mo intervals for 18 mo	Lambda-cyhalothrin, housing improvement, and both combined	Ti, Ts	Paraguay (Humid Chaco)	Infestation rates, 185 DUs initially	Houses: pre-treatment: 42.3% infested; post-treatment: insecticide alone 2.4%, insecticide+housing 16.4%, housing improvement alone 3.4% (all effects reported as significant after McNemar tests). Peridomiciles: initially 13.9%; after treatments, 0%, 3.5%, and 1.7%, respectively (only the combined treatment reported as significant after McNemar tests). Infestation recovered to >6% in 18 mo. Housing improvement costs were >24 times higher than those of insecticide
[98]	Time-series (1977–2000)	Mainly pyrethroids	Mainly Ti, Ts	Paraguay (Dry and Humid Chaco, Alto Paraná Atlantic Forests, Paraná Flooded Savanna)	DU infestation rates	Overall infestation fell from 39.5% (1977) to 14% (1985) and 10% (1996); assessment of ∼170,000 DUs in 1999–2001 yielded an infestation rate of 0.73%, but much higher rates (∼37%) are common in indigenous communities of the Chaco
[99]	Before-after (2 surveys)	National control programme	Ti*, Trv	Uruguay (Uruguayan Savanna)	Infestation rates, ∼240,000 DUs	Pre-intervention: overall rate 2.4% (up to 6.3% in 1 department); by 1992, overall 0.5% (up to 2.7%); by 1999, overall rate 0.1% (up to 0.7%). Ti was virtually eradicated from the country
[100]	Before-after	HCH (2 rounds)	Ti*	Chile (Valdivian Temperate Forests, Chilean Matorral)	Infestation rates, >32,700 DUs in 199 localities	Observed infestation: pre-treatment 3%, post-treatment 0.3%. Infestation as reported by dwellers: pre-treatment 18.7%, post-treatment 3%
[101]	Time-series (1982–95)	“Pyrethroids”, with no specification	Ti*	Chile (Chilean Matorral)	DU infestation rates, ∼480 DUs per assessment	Pre-intervention (1982): 73.3% of DUs infested; 1992, 24.1%; 1993, 3.9%; 1994, 2.8%; 1995, 4%. (Community-based surveillance started in 1992 after a massive spraying campaign [1988–91])
[102]	Before-after	National control programme	Ti*	Chile (Valdivian Temperate Forests, Chilean Matorral)	DU infestation rates	Pre-intervention overall rate was >35% in 1980; systematic control and surveillance from 1993 onwards reduced infestation to <0.5% in 2000. (No information on number of DUs studied each year)
[71]	Before plus 1-yr follow- up	Deltamethrin	Ti	Bolivia (Central Andean Puna and Dry Puna, Bolivian Montane Dry Forests)	Infestation rates, 104 DUs initially	Pre-treatment DU infestation, 77.9%; 6 mo later, 0%; 1 yr post-intervention 6 DUs infested (6.7%), 5 of them with peridomestic colonies only. Assuming independence: OR 0.03, 95%CI 0.01–0.06. Costs estimated as ∼100US$/DU (in 1994–95), including spraying and house improvement. (14 DUs lost in follow-up)
[103]	Time-series (1981–2001)	Mainly Deltamethrin	Mainly Ti	Bolivia (several Andean ecoregions and Chaco)	Infestation rates	DU infestation between 37% and 82% before interventions; rates typically fall to 2–5% after insecticide spraying. Housing improvement alone approximately halves DU infestation rates (e.g., from 60% to 34%)
[104]–[107]	Time-series	Insecticide, surveillance, housing improvements	Mainly Rp	Venezuela (Venezuelan Andes Montane Forests, Llanos, La Costa Xeric Shrublands, Lara-Falcón Dry Forests, Cordillera la Costa Montane Forests)	Infestation rates	Pre-intervention DU infestation rates 60% to 80%; initial Dieldrin spraying in the 50s reduced rates by ∼95%; HCH, Fenitrothion, Propoxur, Deltamethrin, and Lambacyhalothrin were used later; DU infestation fell to 0–40% (late 60s) and then to 1.6–4% (1990–98). Áñez et al. [105] found vectors in ∼70% of 140 DUs in which Chagas disease cases had been diagnosed (1988–2003); they suggest that the disease may be re-emerging as control activities relax. Feliciangeli et al. [107] found triatomines in 24% of 550 DUs surveyed in 2002 and 2004
[61]	Spray all houses vs. only infested	Cyfluthrin	Td	Nicaragua (Central American Dry, Pine-Oak, and Atlantic Moist Forests)	Infestation rates, 188 DUs initially, 260 DUs 1 yr later	Only 1 of 70 DUs found infested initially and sprayed was infested 1 yr later; of 190 DUs not sprayed, 10 were found infested after 1 yr. This apparent difference in infestation rates 1 yr after spraying was however not significant (OR 0.26, 95%CI 0.03–2.08)
[44]	Spraying vs. paints vs. fumigant canisters, with control area	Fenitrothion (spray), probably Malathion in paints and Diclorvos in canisters	Mainly Td, Rp*, also Tnit	Honduras (Central American Dry, Pine-Oak, and Atlantic Moist Forests)	Infestation rates, 750 DUs (150 per group plus 300 control)	At baseline, 35% of DUs (131) infested with Rp, 53% (199) with Td. After 18 mo, 162 DUs were reinfested (134 by Td, 3 by Rp, 1 by both, and 3 by Tnit). In the control area, 49 DUs (16.3%) were infested after 18 mo. The post-treatment odds of infestation were actually *higher* in all treatments than in the control area, particularly among DUs treated with fumigant canisters (OR 2.88, 95%CI 1.83–4.53), which performed significantly worse than any of the alternatives
[108]	Before-after (6–9 mo)	Deltamethrin or Betacyfluthrin	Td, Rp*	Guatemala (Central American Dry and Pine-Oak Forests)	Infestation rates, 947 DUs initially, 923 post-spraying	Pre-intervention: overall, 14% of DUs infested (8.8% with Rp, 5.3% with Td). Post-intervention: 0.8% of DUs infested (0.2% with Rp [2 DUs], 0.5% with Td). Overall OR 0.05, 95%CI 0.02–0.1 (assuming independent observations). Average cost 9.12 US$/DU in 2000. Both insecticides similarly effective
[109]	Before-after (12–16 mo)	Deltamethrin, Lambda-cyhalothrin or Betacyfluthrin	Td	Guatemala (Central American Pine-Oak and Atlantic Moist Forests, Motagua Valley Thornscrub)	Infestation rates, 835 DUs pre- and 1,231 DUs post-intervention	Infestation rates fall from 36% pre- to 8.9% post-intervention. Overall OR 0.18, 95%CI 0.14–0.22 (assuming independent observations)
[110]	Single vs. double vs. triple spraying	Deltamethrin, Lambda-cyhalothrin or Betacyfluthrin	Td	Guatemala (Central American Dry and Pine-Oak Forests)	Infestation rates, from 165 to 1,177 DUs depending on group and assessment round	Single spray round (898–1,177 DUs): infestation falls from 20.8% to 1.4%, but recovers to 8.1% in 20–45 mo. Double spraying (504–765 DUs): baseline infestation (41.9%) falls to 11.9% and 4.8%. Triple spraying (165–242 DUs): initial rate (40.6%) falls to 13.2%, 10.9%, and 4.1% in successive assessments. Pre-trial infestation rates were significantly higher in multiple-spray areas. Overall effect (assuming independent observations): OR 0.17, 95%CI 0.14–0.21
[111]	“Ecosystemic” vs. “traditional”	Deltamethrin +/− housing improvement	Td, Tnit	Guatemala (Central American Dry and Pine-Oak Forests)	Infestation rates, 550 DUs	No significant difference between treatments neither before nor after the intervention. The “ecosystemic” approach (spraying+housing improvement) seems slightly more effective, but costs ∼4 times more than the “traditional” one (insecticide plus basic information)
[112]	Before plus 4 post-intervention assessments	Deltamethrin, Cyfluthrin, Bifenthrin	Tpal, Tbar	Mexico (Balsas Dry Forests)	Infestation rates, 564 DUs	Baseline infestation (15.4%) halves (7%) 1 mo after treatment, then recovers to 10.1% (3 mo), 14.2% (6 mo) and 10.9% (1 yr after treatment). Effect in town centre: McNemar OR (before vs. 1 yr after) 0.75, 95%CI 0.6–1.02; effect in town periphery: McNemar OR (before vs. 1 yr after) 1.3, 95%CI 0.9–1.9
[113]	Before-after	Deltamethrin, Cyfluthrin, Bifenthrin	Tpal	Mexico (Balsas Dry Forests)	Infestation rates, 596 DUs	Infestation rates significantly lower 2 yr after spraying (25% vs. 10.1%; McNemar OR 0.29, 95%CI 0.2–0.4); new infestations were detected in 37 DUs, while infestation disappeared from 126 DUs; 23 DUs were infested both before and after treatment

Ref., reference(s); mo, month(s); yr, year(s); HCH, hexachlorocyclohexane (lindane); Ti, *Triatoma infestans*; Pm, *Panstrongylus megistus*; Ts, *Triatoma sordida*; Tb, *Triatoma brasiliensis*; Tps, *Triatoma pseudomaculata*; Trv, *Triatoma rubrovaria*; Rp, *Rhodnius prolixus*; Td, *Triatoma dimidiata*; Tnit, *Triatoma nitida*; Tpal, *Triatoma pallidipennis*; Tbar, *Triatoma barberi*; an asterisk after the species code indicates that local populations were artificially introduced; the native/introduced status of *T. infestans* in some areas of Paraguay, Argentina, Bolivia, and Chile is dubious. DU, Domiciliary Unit (generally one house and its peridomestic annexes). In the “Setting” column, the ecoregions included in each study are given in parentheses.

The ultimate measure of vector control effectiveness is the reduction of disease incidence. This is usually assessed through serological surveys [116]–[118], with an emphasis on the younger age classes. Domestic triatomine control has resulted in significantly lower seropositivity rates in every country and setting where this has been studied, but residual/re-emerging transmission is not uncommon [6], [18], [63], [97]–[99], [102]–[107], [119]–[126]. Infection rates in vectors [127] and non-human reservoir hosts [74], [128] also decrease sharply in areas under entomological control-surveillance, and this is crucial for reducing household-level disease transmission risk [129].

### Active bug searches versus participatory surveillance

For the purposes of our quantitative appraisal, we defined ‘community participation’ in Chagas disease vector surveillance as simply the involvement of local residents in reporting the presence of suspect bugs in their households. This narrow definition is justified by (i) the need to use some measure of effect size that is (at least qualitatively) comparable across studies, (ii) the fact that vector detection is the primary purpose of entomological surveillance, (iii) the fact than most ‘participatory’ experiences are limited to stimulating bug notification, and (iv) the principle of parsimony, whereby simpler approaches to surveillance, if they are shown to work, enjoy better chances of effectively translating into policy and practice. Table 2 shows the main results of studies quantitatively comparing the effectiveness of vector notification by residents with either active bug searches by control programme staff (the standard approach) or different vector-detection devices (e.g., ‘sensor boxes’) [32], [85], [107], [130]–[132].

**Table 2:** Chagas disease vector surveillance: effectiveness of community involvement in post-control vector detection across regions and triatomine species.

Ref.	Comparison	Vectors	Setting	Units, size	Main results, comments and caveats
[130]	NR vs. ASfo and DDgn	Ti	Chile (Valdivian Temperate Forests, Chilean Matorral)	Detection events in 43 DUs known to be infested by combining the 3 methods	NR vs. ASfo: McNemar OR 0.77, 95%CI 0.3–1.7; ASfo detects Ti in 12 DUs negative by NR, and NR in 9 DUs negative by ASfo. NR vs. DDgn: McNemar OR 0.35, 95%CI 0.15–0.8; 20 DUs negative by NR were positive by DDgn
[85]	NR vs. AS and DDgn	Ti	Brazil (Cerrado)	Detection events in variable DU numbers from 1982 to 1986	NR vs. AS: McNemar OR 6.3, 95%CI 4–10, *N* = 426. NR vs. DDgn: McNemar OR 4.9, 95%CI 2.6–9.2, *N* = 426. (Analyses assume that observations made in different years are independent; year-specific results are congruent, albeit with much larger CIs)
[131]	NR vs. AS and DD	Ti, Ts	Brazil (Cerrado)	Detection events in variable DU numbers from 1984 to 1991	NR vs. AS: McNemar OR 4.2, 95%CI 2.4–6.9, *N* = 269. DD seem to perform relatively poorly: mean annual infestation detected by DD was <20%, vs. >60% by NR. (Unclear data presentation precluded further analyses)
[32]	NR vs. AS	Mainly Ts, Pm	Brazil (Serra do Mar Coastal Forests, Alto Paraná Atlantic Forests, Cerrado)	Detection events in variable DU numbers from 1990 to 1995	Houses: 1990–91, OR 7.2, 95%CI 6.1–8.6, *N*>31,000; 1992–93: OR 5.8, 95%CI 5–6.7, *N*>47,500; 1994–95: OR 4.1, 95%CI 3.5–4.8, *N* = 36,500. Peridomiciles: 1990–91, OR 2.6, 95%CI 2.4–2.9, *N*∼28,000; 1992–93: OR 2.6, 95%CI 2.4–2.8, *N*∼43,500; 1994–95: OR 2.15, 95%CI 1.96–2.4, *N*>33,600. (Analyses assume that all observations are independent, which was likely the case in most instances)
[132]	NR vs. ASfo	Ti	Argentina (Dry Chaco)	Detection events in 98 DUs (1993–96)	Houses: McNemar OR 7, 95%CI 2.1–23.5. Peridomestic areas: McNemar OR 0.2, 95%CI 0.08–0.5 (i.e., ASfo performed better than NR at detecting peridomestic infestation)
[107]	NR vs. AS and DDgn	Mainly Rp, also Tmac, Pg, Rpic	Venezuela (Llanos)	Detection events in 550 DUs	NR vs. AS: McNemar *P*<0.00001. NR vs. DDgn: McNemar OR 127, 95%CI 18–909. This work was performed in areas with low-density vector colonies, with <4 bugs per infested DU. (OR could not be computed for the first comparison because no DU was found to be infested only by AS; 124 infestations were detected only by NR and 7 by both methods. For the NR vs. DDgn comparison, the large OR and CI reflect the fact that infestation was detected only by the devices in only 1 DU, only by NR in 127, and by both methods in 4 DUs)

Ref., reference; NR, notification of vector presence by residents; AS, active searches by vector control staff (ASfo, using a flushing-out agent, generally a low-concentration pyrethroid solution); DD, vector-detection devices (DDgn, Gómez-Núñez boxes); Ti, *Triatoma infestans*; Ts, *Triatoma sordida*; Pm, *Panstrongylus megistus*; Rp, *Rhodnius prolixus*; Tmac, *Triatoma maculata*; Pg, *Panstrongylus geniculatus*; Rpic, *Rhodnius pictipes*. In the “Setting” column, the ecoregions included in each study are given in parentheses.

**Figure 3:**
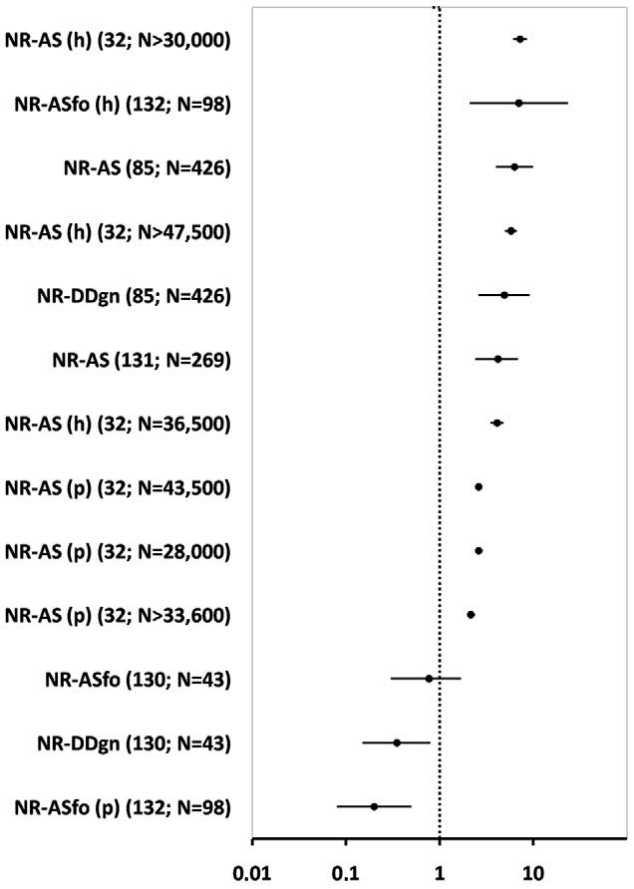
Detection of Chagas disease vectors by notification by residents vs. alternative methods: estimated odds ratios and 95% confidence intervals. NR, notification of vector presence by residents; AS, active searches by vector control staff (ASfo, using a flushing-out agent); DDgn, vector-detection devices (Gómez-Núñez boxes); (h), results regarding bug presence inside houses; (p), results in the peridomestic area; the reference number and sample size are indicated in parentheses; studies were ranked by mean effect size; the vertical dashed line indicates no effect; effects are significant at the 95% level when the CI does not cross the dashed line; point estimate values >1 indicate a positive effect of the first method in the comparison; see Table 2 for details.

With a few exceptions, notification by residents performs obviously much better than active bug searches at detecting infestation foci, although the effect seems to be somewhat smaller in the peridomestic environment [32], [132] (Figure 3). Because notification costs less than active searches, these results are strong indication that it is probably much more cost-effective [20], [116], [133]. Vector-detection devices also seem to be largely outperformed by notification; the evidence is more limited in this case, but comparisons between detection devices and active searches (next subsection) suggest that notification by residents is also superior.

### Vector-detection devices

**Figure 4:**
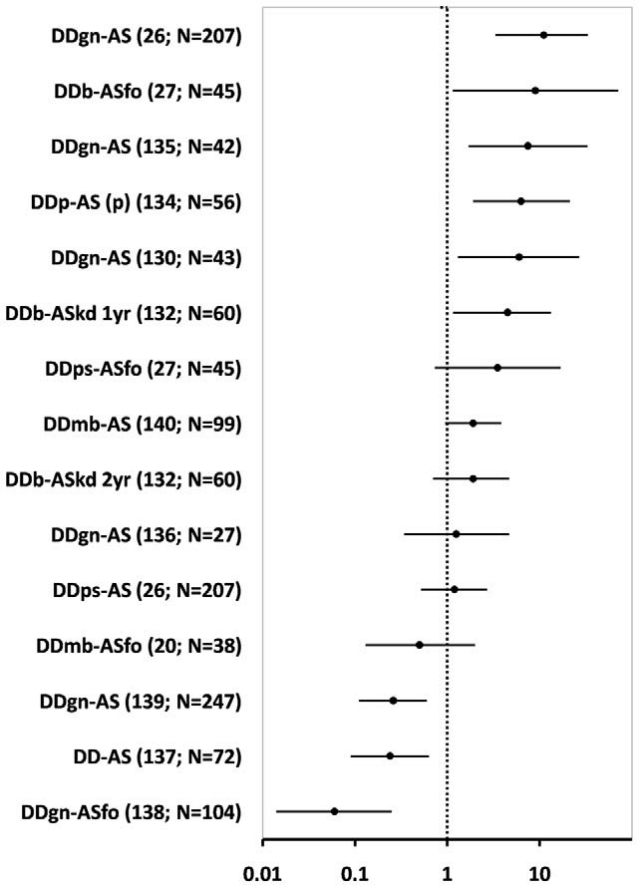
Detection of Chagas disease vectors by vector-detection devices vs. alternative methods: estimated odds ratios and 95% confidence intervals. AS, active searches by vector control staff (ASfo, using a flushing-out agent; ASkd, using full insecticide application to ‘knock-down’ the bugs); DD, vector-detection devices (DDgn, Gómez-Núñez boxes; DDmb, ‘María’ boxes; DDb, box; DDps, paper sheet; DDp, plastic boxes); (p), results in the peridomestic area; the reference number and sample size are indicated in parentheses; studies were ranked by mean effect size; effects are significant at the 95% level when the CI does not cross the dashed line; point estimate values >1 indicate a positive effect of the first method in the comparison; see Table 3 for details.

Several ‘passive’ vector surveillance methods have been devised and tested over the years. As defined here, they differ from the traditional, ‘active’ surveillance approach in that control programme agents do not search the whole residence to determine whether it is infested; instead, they rapidly check for bugs (or their traces) in a ‘detection device’. Table 3 summarises the main results of major comparative studies [20]–[22], [26]–[28], [130], [132], [134]–[140]. In general, the sensitivity of vector-detection devices does not seem to be superior to that of active searches, but (i) both methods appear to complement each other, with only one of them revealing infestation in many instances (see also ref. [141]), and (ii) the costs of the passive approach are, in general, lower (but see ref. [28]). Several studies with small sample sizes favour sensing devices, whereas the results of larger trials tend to show that they perform equally or worse than active searches (Figure 4). The evidence in relation to vector-detection devices remains therefore inconclusive, and further research is needed; below (**Conclusions and outlook**) we provide methodological suggestions to this end.

**Table 3:** Chagas disease vector surveillance: performance of different vector-detection devices across regions and triatomine species.

Ref.	Comparison	Vectors	Setting	Units, size	Main results, comments and caveats
[135]	DDgn vs. AS (nd)	Rp	Venezuela (La Costa Xeric Shrublands, Llanos)	Detection events, 42 DUs, 5 monthly assessments	Overall, DDgn were about 7.5 times more likely to detect infestation than AS (95%CI ∼1.7–33) (Approximate values taken from detection rates averaged over assessments)
[136]	DDgn vs. AS (nc)	Ti	Brazil (Alto Paraná Atlantic Forests, Serra do Mar Coastal Forests)	Detection events, 27 houses and peridomestic annexes	McNemar OR 1.25, 95%CI 0.34–4.7; in 5 cases, only DDgn detected infestation, and in 4 cases only AS did so
[137]	DD vs. AS (nc)	Ts	Brazil (Cerrado)	Detection events, 72 houses and peridomestic annexes	McNemar OR 0.24, 95%CI 0.09–0.63; in 21 cases, only AS detected infestation, and in 5 cases only DD did so
[130]	DDgn vs. AS (c)	Ti	Chile (Valdivian Temperate Forests, Chilean Matorral)	Detection events in 43 DUs known to be infested by combining AS, DDgn and NR	McNemar OR 6, IC95% 1.3–26.8. This positive effect of DDgn on detection only became apparent after several weeks of DDgn operation
[138]	DDgn vs. ASfo (nc/c)	Ti	Brazil (Cerrado, Atlantic Dry Forests)	Detection events, 104 DUs	DDgn performed significantly worse than ASfo: McNemar OR 0.06, 95%CI 0.014–0.25
[139]	DDgn vs. AS (nc/c)	Pm	Brazil (Bahia Interior Forests)	Detection events, 247 DUs	DDgn performed significantly worse than AS: McNemar OR 0.26, 95%CI 0.11–0.6
[20]	DDmb vs. ASfo (nc)	Ti	Argentina (Dry Chaco)	Detection events, 38 DUs	ASfo performed slightly better than DDmb (McNemar OR 0.5, 95%CI 0.13–2); Wisnivesky-Colli et al. [29] suggest that DDmb costs are 4 times lower
[28]	DDmb vs. AS (c)	Ts	Brazil (Atlantic Dry Forests, Caatinga, Cerrado, Bahia Interior Forests)	Detection events, 225 DUs	Infestation rates ascertained with DDmb were about one order of magnitude lower than those reported by control programme agents using AS. AS-based surveillance costs were estimated to be ∼1/4 of those of the DDmb-based strategy, mainly because of the need for several visits per year to check the devices
[21]	DDsf vs. DDmb (nd)	Ti	Argentina (Dry and Humid Chaco)	Detection events, 63 DUs	McNemar OR 14, IC95% 1.8–107. DDsf cheaper than DDmb
[27]	DDb and DDps vs. ASfo (nc)	Ti	Argentina (Dry Chaco)	Detection events, 45 DUs	DDb vs. ASfo: McNemar OR 9, 95%CI 1.14–71. DDps vs. ASfo: OR 3.5, 95%CI 0.73–16.9
[132]	DDb vs. ASkd (c)	Ti	Argentina (Dry Chaco)	Detection events, 60 DUs	After 1 yr of DD operation: McNemar OR 4.5, 95%CI 1.5–13.3. After 2 yr of DD operation: McNemar OR 1.9, 95%CI 0.7–4.7. The results suggested that AS sensitivity depended on vector density – as measured by the number of faecal streaks in DDb. A previous trial [141] suggested that ASfo perform better than ASkd (McNemar OR 5, 95%CI 1.5–17.3), but bug removal by ASfo may have distorted subsequent ASkd results
[134]	DDp vs. AS (nc)	Ti	Argentina (Dry Chaco)	Detection events, 56 peridomestic structures	After 11 mo of DDp operation: McNemar OR 6.3, 95%CI 1.9–21.4. The cost of DDp was also lower
[22]	DDtb vs. AS (nc)	Ti	Argentina (Dry Chaco)	Detection events, 51 peridomestic structures	No differences in performance, but DDtb cost said to be about 12–20% that of AS
[26]	DDgn and DDps vs. AS (nc)	Rec	Peru (Peruvian Yungas, Tumbes-Piura Dry Forests)	Detection events, 207 DUs in 19 localities	DDgn vs. AS: McNemar OR 11.1, 95%CI 3.3–33.3; in 3 DUs infestation was only detected by AS, while in 33 DUs only the DDgn revealed bug presence. DDps and AS were similarly sensitive (McNemar OR 1.2, 95%CI 0.5–2.7) but complemented each other (infestation detected by just one method in 22 DUs)
[140]	DDmb vs. AS (nc)	Mainly Td	Nicaragua (Central American Dry Forests)	Detection events, 99 DUs in 2 communities	DDmb non-significantly more sensitive (McNemar OR 1.9, 95%CI 0.95–3.85); however, AS detect infestation in 12 DUs negative by DDmb

Ref., reference; in the “Comparison” column, letters in parentheses indicate whether the study area was (c) or was not (nc) under chemical vector control; (nc/c) indicates that some, but not all, houses had been recently sprayed, and (nd) that no data on spraying were provided; AS, active searches by vector control staff (ASfo, using a flushing-out agent, generally a low-concentration pyrethroid solution; ASkd, using full insecticide application to ‘knock-down’ the bugs); DD, vector-detection devices (DDgn, Gómez-Núñez boxes; DDmb, ‘María’ boxes; DDsf, ‘Santa Fe’ boxes; DDb, box; DDps, paper sheet; DDp, plastic boxes; DDtb ‘tetra-brick’ recycled boxes; whenever several designs [or an undescribed one] were used, no specification is given); Rp, *Rhodnius prolixus*; Ti, *Triatoma infestans*; Ts, *Triatoma sordida*; Pm, *Panstrongylus megistus*; Rec, *Rhodnius ecuadoriensis*; mo, month(s); yr, year(s). In the “Setting” column, the ecoregions included in each study are given in parentheses.

## Discussion

In the long run, Chagas disease prevention will depend on keeping households free of *T. cruzi* vectors [60], [116], [142]. Insecticide-based control campaigns have been extremely successful, but there is compelling evidence that persistent reinfestation of a fraction of treated households is the pattern to be expected across Latin America; reinfestation, in turn, can result in disease transmission re-emergence [18], [105], [106], [143], [144]. These well-supported findings clearly substantiate the view that long-term vector surveillance will be critical for the interruption of Chagas disease transmission [5], [7], [18], [35], [142], [145], [146].

Entomological surveillance primarily aims at detecting (then eliminating) household infestation foci; it thus allows for monitoring reinfestation trends in areas under control [5], [92], [94], [95], [147]–[151]. This is of fundamental importance for both (i) eliminating residual foci of introduced species targeted for local eradication and (ii) keeping reinfestation by native species at levels below disease transmission thresholds [73], [115], [152], [153]. We note, however, that ‘native’ vector species may be equally or more efficient than introduced ones at transmitting *T. cruzi*, and that even the most notorious ‘primary’ vectors, *T. infestans* and *Rhodnius prolixus*, are native (and reinfest treated households) [18], [143], [154]–[158] in their original ranges. Thus, entomological surveillance has a major role to play in most of Latin America even after introduced vector populations have been eliminated; in areas under surveillance, rapid diagnostic tests could be used to discover residual or re-emergent transmission foci [142].

But in order to attain these goals, vector detection must be as effective as possible, and the evidence we have reviewed shows that available vector-detection techniques all work far from perfectly. What would be, then, the best strategy to meet the permanent challenge of detecting reinfestation? Our appraisal yields strong support to the view that notification of suspect vectors by residents is the most sensitive among the several detection approaches tested to date – and that it is also probably the cheapest. Furthermore, the difference in performance seems to widen as vector population density declines, which is the typical situation in post-control settings.

Such an austere ‘participatory’ strategy signals the minimum degree of community involvement required to effectively enhance surveillance: residents are just asked to report suspect insects found in their homes, and a response is mounted by professional staff, often related to decentralised health services [142], [154], [159], [160], to eliminate infestation when needed [18], [145], [161]. An educational/communication component tailored to the social-cultural background of the community is obviously required to stimulate notification [4], [35], [162], [163], but our review suggests that very simple interventions can be effective enough. Perhaps the main challenge here is to sustain community awareness in the face of even rarer infestation events; continuous education, a clearly defined channel for communication between residents and control agents, and an opportune response to any notification (including those involving insects other than triatomines) are probably the key to long-term success [35], [73], [152], [159], [164]–[166].

This is not to say that more sophisticated approaches would not perhaps bring further benefits to people living under risk conditions. For instance, we found that most community-based experiences in Chagas disease vector surveillance are merely utilitarian, with little or no participation of the community in the design, planning, and evaluation of interventions. Effective involvement of all stakeholders along the whole process would no doubt foster true empowerment, and this could in itself result in improved health and living standards [33], [34], [167]–[171]. Still, we underscore that, in the absence of adequate resources for comprehensive community-based programmes, stimulating vector notification by residents may suffice to boost the efficiency of entomological surveillance across highly diverse ecological and socio-economic settings.

Finally, our review revealed that there is plenty of room for improvement of both methodological and reporting standards in the Chagas disease control/surveillance literature. In many cases, the results were reported incompletely and/or confusingly, sometimes precluding data extraction; in several instances, the data in the text, tables, and figures were incongruent. Indeed, just a few of the reviewed studies followed high-quality designs (e.g., with some sort of randomisation) and used sound analytical approaches, particularly in relation to the non-independence of observations; these reports tended to rely on small sample sizes and/or have limited spatial scope. Apart from the obvious need for using adequate design and analytical procedures, several guidelines for good reporting practices are readily available (e.g., the STROBE statement [172]); researchers and journal editors share the responsibility of improving the standards of published reports on Chagas disease control and prevention.

Indeed, we believe that the main limitations of our review relate to the quality of the original reports, even if the breadth of our appraisal probably lightens the effects of individual study drawbacks. We did not test formally for publication bias, but deem it unlikely that any major study was overlooked; the possibility that such a bias exists should however be kept in mind when interpreting our results, particularly in relation to vector-detection devices. In an attempt to overcome possible study-level biases, we made every effort to extract and re-analyse the data in each document, without taking reported results at face value, but this does not alleviate design or data collection bias. However, we are confident that our main findings (that reinfestation by triatomines is common and widespread and that householder involvement in vector reporting enhances surveillance) are not bias-induced artefacts. We also note that our assessment focused on the initial stage of surveillance – the detection of infestation foci. The responses triggered by detection events, the monitoring of infestation trends, and the analysis and dissemination of epidemiological data are also essential components of disease surveillance [173], but their appraisal was beyond the scope of this review.

### Conclusions and outlook

Entomological surveillance is and will remain crucial to contain Chagas disease transmission; yet, the zoonotic nature of the parasite's life cycle implies that eradication is unfeasible [1]. The enduring challenge of household reinfestation by locally native vectors can only be met by means of horizontal strategies – and these work better when the community takes on a protagonist role. Even very simple forms of participation, such as encouraging vector notification by residents, can substantially enhance the effectiveness of surveillance. Control programmes should therefore incorporate community-based approaches as a strategic asset from inception; such approaches must include a timely, professional response to every notification, and would very likely benefit from a strengthened focus on community empowerment.

It must finally be emphasised that, in practice, vector detection failures are unavoidable, particularly when bug population density is low [174]. It may then be argued that infestation rates are virtually always underestimated and that, because these rates are the foremost indicator used in decision-making [175], imperfect detection can seriously misguide Chagas disease control programme management. We consequently suggest that a critical area for future research relates to the reliable estimation of vector detection probabilities. This is somewhat more difficult in the absence of a ‘gold-standard’ technique, but by no means unworkable: repeated-sampling approaches [176]–[178] readily yield detection probability estimates (with confidence intervals) that can in addition be modelled as a function of covariates – such as, for instance, alternative detection methods, different fieldwork teams, different vector species, or physically diverse ecotopes. These approaches have been successfully applied in wildlife [179] and disease ecology studies [180], [181], and can also help enhance Chagas disease vector research [182].

## Supporting Information

Abstract S1 Spanish and Portuguese translations of the abstract.
(DOC)

Checklist S1 PRISMA checklist.
(DOC)

List S1 93 documents submitted to full data extraction.
(DOC)

List S2 27 documents used in partial quantitative assessments but not submitted to full data extraction.
(DOC)
