# Supplementary material for: Community Participation in Chagas Disease Vector Surveillance: Systematic Review
Source: PLoS Negl Trop Dis. 2011 Jun 21;5(6):e1207. doi: 10.1371/journal.pntd.0001207 (PMC3119642; doi:10.1371/journal.pntd.0001207)
Supplement: Abstract S1 — Spanish and Portuguese translations of the abstract. (DOC) [file pntd.0001207.s001.doc]

**Resumen**

***Introducción:*** El control de vectores ha reducido sustancialmente la incidencia de la enfermedad de Chagas; con todo, la transmisión mediada por triatominos capaces de reinfestar viviendas persiste. La vigilancia entomológica es, por tanto, crucial, para la interrupción de la transmisión a largo plazo. Sin embargo, los focos de infestación son cada vez más pequeños y difíciles de detectar a medida que el control avanza, haciéndose necesario desarrollar métodos de vigilancia más sensibles. La participación de la comunidad y diversos tipos de aparatos detectores de vectores podrían mejorar el desempeño de la vigilancia, pero no existe, hasta el momento, una evaluación exhaustiva de esta idea.

***Metodología/Resultados Principales:*** Realizamos búsquedas bibliográficas en Medline, Web of Knowledge, Scopus, LILACS, SciELO, las referencias de los estudios recuperados y nuestros propios archivos. Los datos de estudios que describen intervenciones de control y/o vigilancia de vectores fueron extraídos por dos revisores. Los resultados de interés primario incluyeron cambios en las tasas de infestación y la detección de focos de infestación/reinfestación. La probable dependencia de la mayoría de los resultados de las condiciones específicas de cada estudio y localidad impidió la realización de meta-análisis, pero reanalizamos, siempre que nos fue posible, los datos de estudios comparativos sobre métodos de control y detección de vectores. Los resultados confirman que la aplicación de insecticidas por profesionales es altamente efectiva, pero muestran también que la reinfestación por triatominos nativos es frecuente y se extiende por toda América Latina. La notificación de insectos sospechosos por los habitantes (la estrategia participativa más sencilla) incrementa de forma notable las probabilidades de detección de vectores; en comparación, tanto las búsquedas activas cuanto los aparatos detectores tienen un desempeño muy pobre, aunque es posible que se complementen en ciertos casos.

***Conclusiones/Importancia:*** La participación de la comunidad debería convertirse en un componente estratégico de la vigilancia de la enfermedad de Chagas; sin embargo, sólo la aplicación profesional de insecticidas parece ser consistentemente efectiva en la eliminación de focos de infestación. Estas estrategias participativas probablemente mejorarían si se garantizase la participación efectiva de todos los actores en las diferentes fases del proceso, desde la planificación a la evaluación.

**Resumo**

***Introdução:*** O controle de vetores tem reduzido substancialmente a incidência da doença de Chagas; apesar disso, a transmissão mediada por triatomíneos capazes de reinfestar moradias persiste. A vigilância entomológica é, portanto, crucial para a interrupção da transmissão no longo prazo. Contudo, os focos de infestação se tornam progressivamente menores e mais difíceis de detectar à medida que o controle avança, fazendo necessário o desenvolvimento de métodos de vigilância mais sensíveis. A participação da comunidade e diversos tipos de aparelhos detectores de vetores poderiam melhorar o desempenho da vigilância, mas ainda não existe uma avaliação exaustiva desta idéia.

***Metodologia/Resultados Principais:*** Realizamos buscas bibliográficas em Medline, Web of Knowledge, Scopus, LILACS, SciELO, as referências dos estudos recuperados e os nossos arquivos. Os dados de estudos que descrevem intervenções de controle e/o vigilância vetorial foram extraídos por dois revisores. Os resultados de interesse primário incluíram mudanças nas taxas de infestação e detecção de focos de infestação/reinfestação. A provável dependência da maioria dos resultados das condições específicas de cada estudo e localidade impediu a realização de meta-análises, mas re-analisamos, sempre que foi possível, os dados de estudos comparativos sobre métodos de controle e detecção de vetores. Os resultados confirmam que a aplicação de inseticidas por profissionais é altamente efetiva, mas mostram também que a reinfestação por triatomíneos nativos é freqüente e se estende por toda a América Latina. A notificação de insetos suspeitos por parte dos moradores (a estratégia participativa mais simples) aumenta de forma notável as probabilidades de detecção de vetores; em comparação, tanto as buscas ativas quanto os aparelhos detectores têm um desempenho muito pobre, embora seja possível que se complementem em certos casos.

***Conclusões/Importância:*** A participação da comunidade deveria se converter em um componente estratégico da vigilância da doença de Chagas; contudo, só a aplicação profissional de inseticidas parece ser consistentemente efetiva na eliminação de focos de infestação. Estas estratégias participativas provavelmente melhorariam se a participação efetiva de todos os atores nas diferentes fases do processo, desde o planejamento até a avaliação, fosse garantida.
