# Supplementary material for: Community Participation in Chagas Disease Vector Surveillance: Systematic Review
Source: PLoS Negl Trop Dis. 2011 Jun 21;5(6):e1207. doi: 10.1371/journal.pntd.0001207 (PMC3119642; doi:10.1371/journal.pntd.0001207)
Supplement: List S1 — 93 documents submitted to full data extraction. (DOC) [file pntd.0001207.s003.doc]

**Supporting Information**

**List S1: 93 documents submitted to full data extraction**

Acevedo F, Godoy E, Schofield CJ (2000) Comparison of intervention strategies for control of *Triatoma* *dimidiata* in Nicaragua. Mem Inst Oswaldo Cruz 95(6): 867–871.

Aché A, Matos AJ (2001) Interrupting Chagas disease transmission in Venezuela. Rev Inst Med Trop São Paulo 43(1): 37–43.

Ávila Montes GA, Ponce C, Ponce E, Martínez Hernández M, Flores M (1999) Insecticidal paint and fumigant canisters for Chagas’ disease control: community acceptance in Honduras. Rev Panam Salud Pública 6(5): 311–320.

Campbell-Lendrum DH, Angulo VM, Esteban L, Tarazona Z, Parra GJ, et al. (2007) House-level risk factors for triatomine infestation in Colombia. Int J Epidemiol 36(4): 866–872.

Candioti C, Paulone I (1997) Detector de vinchucas Santa Fe: un diseño de alta sensibilidad y bajo costo. Medicina (B Aires) 57(4): 433–436.

Cardinal MV, Castañera MB, Lauricella MA, Cécere MC, Ceballos LA, et al. (2006) A prospective study of the effects of sustained vector surveillance following community-wide insecticide application on *Trypanosoma cruzi* infection of dogs and cats in rural Northwestern Argentina. Am J Trop Med Hyg 75(4): 753–761.

Cardinal MV, Lauricella MA, Marcet PL, Orozco MM, Kitron U, et al. (2007) Impact of community-based vector control on house infestation and *Trypanosoma cruzi* infection in *Triatoma infestans*, dogs and cats in the Argentine Chaco. Acta Trop 103(3): 201–211.

Carneiro M (2002) Estudos epidemiológicos na avaliação de efetividade do Programa de Controle da doença de Chagas: discussão metodológica. Rev Bras Epidemiol 5(1): 129–141.

Carneiro M, Antunes C (1994) Avaliação de eficácia do Programa de Controle da doença de Chagas: aspectos metodológicos. Cad Saúde Pública 10(Suppl.): S261–S272.

Carvalho ME, Silva RA, Rodrigues VLCC, Oliveira CD (2002) Programa de Controle da doença de Chagas no Estado de São Paulo: sorologia de moradores como parte de investigação de unidades domiciliares com presença de triatomíneos vetores na década de 1990. Cad Saúde Pública 18(6): 1695–1703.

Cécere MC, Castañera MB, Canale DM, Chuit R, Gürtler RE (1999) *Trypanosoma cruzi* infection in *Triatoma infestans* and other triatomines: long-term effects of a control program in rural northwestern Argentina. Rev Panam Salud Pública 5(6): 392–399.

Cécere MC, Gürtler RE, Canale D, Chuit R, Cohen JE (1997) The role of the peridomiciliary area in the elimination of *Triatoma infestans* from rural Argentine communities. Rev Panam Salud Pública 1(4): 273–279.

Cécere MC, Gürtler RE, Canale DM, Chuit R, Cohen JE (2002) Effects of partial housing improvement and insecticide spraying on the reinfestation dynamics of *Triatoma infestans* in rural northwestern Argentina. Acta Trop 84(2): 101–116.

Cécere MC, Vázquez-Prokopec GM, Gürtler RE, Kitron U (2004) Spatio-temporal analysis of reinfestation by *Triatoma infestans* (Hemiptera: Reduviidae) following insecticide spraying in a rural community in northwestern Argentina. Am J Trop Med Hyg 71(6): 803–810.

Cécere MC, Vázquez-Prokopec GM, Gürtler RE, Kitron U (2006) Reinfestation sources for Chagas disease vector, *Triatoma infestans*, Argentina. Emerg Infect Dis 12(7): 1096–1102.

Cedillos RA (1998) The effectiveness of design and construction materials in Chagas’ disease vector control. Rev Argent Microbiol 20(Suppl.1): 53–57.

Chuit R, Paulone I, Wisnivesky-Colli C, Bo R, Pérez AC, et al. (1992) Result of a first step toward community-based surveillance of transmission of Chagas’ disease with appropriate technology in rural areas. Am J Trop Med Hyg 46(4): 444–450.

Cohen JM, Wilson ML, Cruz-Celis A, Ordóñez R, Ramsey JM (2006) Infestation by *Triatoma* *pallidipennis* (Hemiptera: Reduviidae: Triatominae) is associated with housing characteristics in rural Mexico. J Med Entomol 43(6): 1252–1260.

Cuba Cuba CA, Vargas F, Roldán J, Ampuero C (2003) Domestic *Rhodnius ecuadoriensis* (Hemiptera, Reduviidae) infestation in Northern Peru: a comparative trial of detection methods during a six-month follow-up. Rev Inst Med Trop São Paulo 45(2): 85–90.

de Marco RJ, Gürtler RE, Salomón OD, Chuit R (1999) Small-scale field trial of a sensing device for detecting peridomestic populations of *Triatoma infestans* (Hemiptera: Reduviidae) in northwestern Argentina. J Med Entomol 36(6): 884–887.

Dias JCP (1968) Reinfestação do município de Bambuí por triatomíneos transmissores da doença de Chagas (2ª nota). Mem Inst Oswaldo Cruz 66(2): 197–208.

Dias JCP, Vieira EP, Tadashi H, Azeredo BVM (2005) Nota sobre o uso de bio-sensores "Maria" nas ações de vigilância entomológica contra a doença de Chagas ao norte de Minas Gerais. Rev Soc Bras Med Trop 38(5): 377–382.

Diotaiuti L, de Paula OR, Falcão PL, Dias JCP (1994) Evaluation of the Chagas’ disease vector control program in Minas Gerais, Brazil, with special reference to *Triatoma* *sordida*. Bull Pan Am Health Organ 28(3): 211–219.

Diotaiuti L, Faria Filho OF, Carneiro FCF, Dias JCP, Pires HHR, et al. (2000) Aspectos operacionais do controle do *Triatoma* *brasiliensis*. Cad Saúde Pública 16(Suppl.2): 61–67.

Dumonteil E, Ramírez-Sierra MJ, Ferral J, Euan-García M, Chavez-Nuñez L (2009) Usefulness of community participation for the fine temporal monitoring of house infestation by non-domiciliated triatomines. J Parasitol 95(2): 469–471.

Dumonteil E, Ruiz Piña H, Rodríguez-Félix E, Barrera-Pérez M, Ramírez-Sierra MJ, et al. (2004) Re-infestation of houses by *Triatoma* *dimidiata* after intra-domicile insecticide application in the Yucatán peninsula, Mexico. Mem Inst Oswaldo Cruz 99(3): 253–256.

Falavigna-Guilherme AL, Costa AL, Batista O, Pavanelli GC, Araújo SM (2002) Atividades educativas para o controle de triatomíneos em área de vigilância epidemiológica do Estado do Paraná, Brasil. Cad Saúde Pública 18(6): 1543–1550.

Feliciangeli MD, Hernández M, Suárez B, Martínez C, Bravo A, et al. (2007) Comparación de métodos de captura intradoméstica de triatominos vectores de la enfermedad de Chagas en Venezuela. Bol Dir Malar Salud Amb 47(1): 103–117.

Ferro EA, Rojas de Arias A, Ferreira ME, Simancas LC, Rios LS, et al. (1995) Residual effect of lambdacyhalothrin on *Triatoma infestans*. Mem Inst Oswaldo Cruz 90(3): 415–419.

Fleming-Moran M (1992) The initial success of the Chagas’ disease control program: factors contributing to *Triatomine* infestation. Cad Saúde Pública 8(4): 391–403.

Forattini OP, Ferreira OA, Rabello EX, Barata JMS, Santos JLF (1983) Aspectos ecológicos da tripanossomíase americana. XIX – Desenvolvimento da domiciliação triatomínea regional, em centro de endemismo de *Panstrongylus megistus*. Rev Saúde Pública 17(6): 436–460.

Forattini OP, Ferreira OA, Rabello EX, Barata JMS, Santos JLF (1983) Aspectos ecológicos da tripanossomíase americana. XVII – Desenvolvimento da domiciliação triatomínea regional, em centro de endemismo de *Triatoma sordida*. Rev Saúde Pública 17(3): 159–199.

Forattini OP, Ferreira OA, Souza JMP, Rabello EX, Rocha e Silva EO, et al. (1973) Medida da infestação domiciliar por *Triatoma* *sordida*. Rev Saúde Pública 7(3): 241–250.

Forattini OP, Juarez E, Corrêa RR (1969) Medida da infestação domiciliar por *Triatoma* *infestans*. Rev Saúde Pública 3(1): 11–16.

García-Zapata MT, Marsden PD (1992) Control of the transmission of Chagas’ disease in Mambaí, Goiás, Brazil (1980-1988). Am J Trop Med Hyg 46(4): 440–443.

García-Zapata MT, Marsden PD (1993) Chagas’ disease: control and surveillance through use of insecticides and community participation in Mambaí, Goiás, Brazil. Bull Pan Am Health Organ 27(3): 265–279.

García-Zapata MT, Marsden PD, Virgens D, Soares VA (1988) Epidemiological vigilance with community participation in the control of the vectors of Chagas’ disease in Goiás, Central Brazil. Rev Argent Microbiol 20(Suppl.1): 106–117.

Gómez-Núñez JC (1965) Desarrollo de un nuevo método para evaluar la infestación intradomiciliaria por *Rhodnius prolixus*. Acta Cient Venez 16(1): 26–31.

González J, Contreras MC, Schenone H, Adaos H, Cabezas R (1996) Enfermedad de Chagas: Impacto del programa de control del *Triatoma infestans* en la comuna de Alto del Carmen, provincia de Huasco, III región Atacama, Chile. Bol Chil Parasitol 51(1-2): 28–30.

Guillén G, Díaz R, Jemio A, Alfred Cassab J, Teixeira Pinto C, et al. (1997) Chagas disease vector control in Tupiza, southern Bolivia. Mem Inst Oswaldo Cruz 92(1): 1–8.

Gürtler RE, Canale DM, Spillmann C, Stariolo R, Salomón OD, et al. (2004) Effectiveness of residual spraying of peridomestic ecotopes with deltamethrin and permethrin on *Triatoma infestans* in rural western Argentina: a district-wide randomized trial. Bull World Health Organ 82(3): 196–205.

Gürtler RE, Cécere MC, Canale DM, Castañera MB, Chuit R, et al. (1999) Monitoring house reinfestation by vectors of Chagas disease: a comparative trial of detection methods during a four-year follow-up. Acta Trop 72(2): 213–234.

Gürtler RE, Chuit R, Cécere MC, Castañera MB (1995) Detecting domestic vectors of Chagas disease: a comparative trial of six methods in north-west Argentina. Bull World Health Organ 73(4): 487–494.

Gürtler RE, Kitron U, Cécere MC, Segura EL, Cohen JE (2007) Sustainable vector control and management of Chagas disease in the Gran Chaco, Argentina. Proc Natl Acad Sci USA 104(41): 16194–16199.

Gürtler RE, Petersen RM, Cécere MC, Schweigmann NJ, Chuit R, et al. (1994) Chagas disease in north-west Argentina: risk of domestic reinfestation by *Triatoma infestans* after a single community-wide application of deltamethrin. Trans R Soc Trop Med Hyg 88(1): 27–30.

Gürtler RE, Schweigmann NJ, Cécere MC, Chuit R, Wisnivesky-Colli C (1993) Comparison of two sampling methods for domestic populations of *Triatoma infestans* in north-west Argentina. Med Vet Entomol 7(3): 238–242.

Gürtler RE, Vázquez-Prokopec GM, Ceballos LA, Petersen CL, Salomón OD (2001) Comparison between two artificial shelter units and timed manual collections for detecting peridomestic *Triatoma infestans* (Hemiptera: Reduviidae) in rural northwestern Argentina. J Med Entomol 38(3): 429–436.

Hashimoto K, Cordón-Rosales C, Trampe R, Kawabata M (2006) Impact of single and multiple residual sprayings of pyrethroid insecticides against *Triatoma* *dimidiata* (Reduviidae; Triatominae), the principal vector of Chagas disease in Jutiapa, Guatemala. Am J Trop Med Hyg 75(2): 226–230.

Herber O, Kroeger A (2003) Pyrethroid-impregnated curtains for Chagas’ disease control in Venezuela. Acta Trop 88(1): 33–38.

Jörg ME (1989) La modificación del biotopo perihabitacional en la profilaxis de la enfermedad de Chagas. Rev Soc Bras Med Trop 22(2): 91–95.

Kroeger A, Villegas E, Ordóñez-González J, Pabon E, Scorza JV (2003) Prevention of the transmission of Chagas’ disease with pyrethroid-impregnated materials. Am J Trop Med Hyg 68(3): 307–311.

Lima VLC, Yaguchi MK, Alves ZCPVT (1990) Aspectos da atividade de "notificação de barbeiros" pela população no controle de *Panstrongylus megistus* em 12 municípios da região Nordeste do estado de São Paulo, Brasil, 1974 a 1983. Rev Saúde Pública 24(6): 497–505.

Lorca M, García A, Contreras MC, Schenone H, Rojas A (2001) Evaluation of a *Triatoma infestans* elimination program by the decrease of *Trypanosoma cruzi* infection frequency in children younger than 10 years, Chile, 1991-1998. Am J Trop Med Hyg 65(6): 861–864.

Lorca M, Schenone H, Contreras MC, García A, Rojas A, et al. (1996) Evaluación de los programas de erradicación de vectores de la enfermedad de Chagas en Chile mediante estudio serológico de niños menores de 10 años. Bol Chil Parasitol 51(3-4): 80–85.

Maluf J, Guarita OF, Rocha e Silva EO (1970) O contrôle da doença de Chagas no município de Bariri, Estado de São Paulo. Rev Saúde Pública 4(1): 7–12.

Marcondes CB (1989) Eficiência de alfacipermetrina e cipermetrina no controle de triatomíneos em Camalaú, no sul da Paraíba (Hemiptera: Reduviidae). Mem Inst Oswaldo Cruz 84(Suppl.IV): 343–347.

Monroy C, Bustamante DM, Pineda S, Rodas A, Castro X, et al. (2009) House improvements and community participation in the control of *Triatoma* *dimidiata* re-infestation in Jutiapa, Guatemala. Cad Saúde Pública 25(Suppl.1): S168–S178.

Nakagawa J, Cordón-Rosales C, Juárez J, Itzep C, Nonami T (2003) Impact of residual spraying on *Rhodnius* *prolixus* and *Triatoma* *dimidiata* in the department of Zacapa in Guatemala. Mem Inst Oswaldo Cruz 98(2): 277–281.

Nakagawa J, Hashimoto K, Cordón-Rosales C, Juárez JA, Trampe R, et al. (2003) The impact of vector control on *Triatoma* *dimidiata* in the Guatemalan department of Jutiapa. Ann Trop Med Parasitol 97(3): 288–297.

Neghme A, Schenone H, Villaroel F, Rojas A (1991) Programa antitriatómico experimental de Santiago. Bol Chil Parasitol 46(3-4): 47–57.

Oliveira Filho AM (1996) Uso de nuevas herramientas para el control de triatominos en diferentes situaciones entomológicas en el continente americano. Rev Soc Bras Med Trop 30(1): 41–46.

Oliveira Filho AM, Melo MT, Santos CE, Faria Filho OF, Carneiro FCF, et al. (2000) Tratamentos focais e totais com inseticidas de ação residual para o controle de *Triatoma* *brasiliensis* e *Triatoma* *pseudomaculata* no Nordeste brasileiro. Cad Saúde Pública 16(Suppl.2): 105–111.

Oliveira-Lima JW, Faria Filho OF, Vieira JBF, Gadelha FV, Oliveira Filho AM (2000) Alterações do peridomicílio e suas implicações para o controle do *Triatoma* *brasiliensis*. Cad Saúde Pública 16(Suppl.2): 75–81.

Palma-Guzmán R, Rivera R, Morales G (1996) Domestic vectors of Chagas’ disease in three rural communities of Nicaragua. Rev Inst Med Trop São Paulo 38(2): 133–140.

Passos AD, Nogueira JL, Figueiredo JFC, Gomes UA, Dal-Fabbro AL (1997) Evolução da positividade sorológica para a doença de Chagas numa comunidade rural brasileira. Rev Panam Salud Pública 2(4): 247–252.

Paulone I, Chuit R, Pérez A, Wisnivesky-Colli C, Segura E (1988) Field research on an epidemiological surveillance alternative of Chagas’ disease transmission: The primary health care (PHC) strategy in rural areas. Rev Argent Microbiol 20(Suppl.1): 103–105.

Piesman J, Sherlock IA (1984) Sensitivity of Gómez-Núñez boxes for the detection of household infestation with *Panstrongylus megistus*. Rev Soc Bras Med Trop 17(1): 17–20.

Pinchin R, Fanara DM, Castleton CW, Oliveira Filho AM (1981) Comparison of techniques for detection of domestic infestations with *Triatoma infestans* in Brazil. Trans R Soc Trop Med Hyg 75(5): 691–694.

Pinchin R, Oliveira Filho AM, Gilbert B (1981) Field trial of permethrin for the control of *Triatoma infestans*. Bull Pan Am Health Organ 15(4): 370–376.

Porcasi X, Catalá SS, Hrellac H, Scavuzzo MC, Gorla DE (2006) Infestation of rural houses by *Triatoma infestans* (Hemiptera: Reduviidae) in southern area of Gran Chaco in Argentina. J Med Entomol 43(5): 1060–1067.

Ramsey JM, Cruz-Celis A, Salgado L, Espinosa L, Ordóñez R, et al. (2003) Efficacy of pyrethroid insecticides against domestic and peridomestic populations of *Triatoma* *pallidipennis* and *Triatoma barberi* (Reduviidae: Triatominae) vectors of Chagas’ disease in Mexico. J Med Entomol 40(6): 912–920.

Rocha e Silva EO, Maluf J, Corrêa RR (1970) Doença de Chagas: atividades de vigilância entomológica numa área do Estado de São Paulo, Brasil. Rev Saúde Pública 4(2): 129–145.

Rocha e Silva EO, Wanderley DM, Rodrigues VLCC (1998) *Triatoma infestans*: importância, controle e eliminação da espécie no estado de São Paulo, Brasil. Rev Soc Bras Med Trop 31(1): 73–88.

Rojas de Arias A, Ferro EA, Ferreira ME, Simancas LC (1999) Chagas disease vector control through different intervention modalities in endemic localities of Paraguay. Bull World Health Organ 77(4): 331–339.

Schenone H, Villarroel F, Rojas A, Carrasco J (1979) Estudio comparativo del rendimiento de tres métodos de detección de la presencia de *Triatoma infestans* en viviendas infestadas. Bol Chil Parasitol 34(1): 7–12.

Segura EL, Cura EN, Sosa Estani SA, Andrade J, Lansetti JC, et al. (2000) Long-term effects of a nationwide control program on the seropositivity for *Trypanosoma cruzi* infection in young men from Argentina. Am J Trop Med Hyg 62(3): 353–362.

Silva RA, Bonifácio PR, Wanderley DMV (1999) Doença de Chagas no estado de São Paulo: comparação entre pesquisa ativa de triatomíneos em domicílios e notificação de sua presença pela população em área sob vigilância entomológica. Rev Soc Bras Med Trop 32(6): 653–659.

Silva RA, Scandar SA, Pauliquévis-Júnior C, Sampaio SMP, Rodrigues VLCC (2005) Ampliação de raio de pesquisa de triatomíneos na atividade de atendimento às notificações em área de *Triatoma* *sordida* (Stål, 1859) no estado de São Paulo. Rev Soc Bras Med Trop 38(4): 339–343.

Silva RA, Wanderley DM, Domingos MF, Yasumaro S, Scandar SAS, et al. (2006) Doença de Chagas: notificação de triatomíneos no estado de São Paulo na década de 1990. Rev Soc Bras Med Trop 39(5): 488–494.

Silveira AC, Peñarada-Carrillo R, Lorosa ES, Leite J, Vinhaes MC, et al. (2001) Evaluation of the impact of chemical control measures and entomological surveillance on Chagas’ disease in the counties of Mambaí and Buritinópolis, Goiás State, Brazil. Rev Soc Bras Med Trop 34(6): 549–557.

Sosa Estani S, Dri S, Touris C, Abalde S, Dell’Arciprete A, et al. (2009) Transmisión vectorial y congénita de *Trypanosoma cruzi* en Las Lomitas, Formosa. Medicina (B Aires) 69(4): 424–430.

Tonn RJ, Otero RA, Jiménez J (1976) Comparación del método hora-hombre con la trampa Gómez-Núñez en la búsqueda de *Rhodnius prolixus*. Bol Dir Malariol Salud Ambient 16(3): 269–275.

Vázquez-Prokopec GM, Ceballos LA, Salomón OD, Gürtler RE (2002) Field trials of an improved cost-effective device for detecting peridomestic populations of *Triatoma infestans* (Hemiptera: Reduviidae) in rural Argentina. Mem Inst Oswaldo Cruz 97(7): 971–977.

Vázquez-Prokopec GM, Cécere MC, Canale DM, Gürtler RE, Kitron U (2005) Spatiotemporal patterns of reinfestation by *Triatoma guasayana* (Hemiptera: Reduviidae) in a rural community of northwestern Argentina. J Med Entomol 42(4): 571–581.

Villela MM, Souza JB, Mello VP, Azeredo BVM, Dias JCP (2005) Vigilância entomológica da doença de Chagas na região centro-oeste de Minas Gerais, Brasil, entre os anos de 2000 e 2003. Cad Saúde Pública 21(3): 878–886.

Villela MM, Souza JMB, Melo VP, Dias JCP (2009) Avaliação do Programa de Controle da Doença de Chagas em relação à presença de *Panstrongylus megistus* na região centro-oeste do estado de Minas Gerais, Brasil. Cad Saúde Pública 25(4): 907–917.

Wanderley DMV (1991) Vigilância entomológica da doença de Chagas no estado de São Paulo. Rev Saúde Pública 25(1): 28–32.

Wastavino GR, Cabrera-Bravo M, García de La Torre G, Vences-Blanco M, Ruiz Hernández A, et al. (2004) Insecticide and community interventions to control *Triatoma* *dimidiata* in localities of the State of Veracruz, Mexico. Mem Inst Oswaldo Cruz 99(4): 433–437.

Wisnivesky-Colli C (1993) La importancia del peridomicilio en un programa de eliminación de *Triatoma infestans*. Rev Soc Bras Med Trop 26(Suppl.3): 55–63.

Wisnivesky-Colli C, Paulone I, Pérez A, Chuit R, Gualtieri J, et al. (1987) A new tool for continuous detection of the presence of triatomine bugs, vectors of Chagas disease in rural households. Medicina (B Aires) 47(1): 45–50.

Zeledón R, Rojas JC (2006) Environmental management for the control of *Triatoma dimidiata* (Latreille, 1811), (Hemiptera: Reduviidae) in Costa Rica: a pilot project. Mem Inst Oswaldo Cruz 101(4): 379–386.

Zeledón R, Rojas JC, Urbina A, Cordero M, Gamboa SH, et al. (2008) Ecological control of *Triatoma dimidiata* (Latreille, 1811): five years after a Costa Rican pilot project. Mem Inst Oswaldo Cruz 103(6): 619–621.

Zerba EN, Wallace G, Picollo MI, Casabé N, de Licastro S, et al. (1997) Evaluación de la β-cipermetrina para el control de *Triatoma infestans*. Rev Panam Salud Pública 1(2): 133–137.
