# Supplementary material for: Community Participation in Chagas Disease Vector Surveillance: Systematic Review
Source: PLoS Negl Trop Dis. 2011 Jun 21;5(6):e1207. doi: 10.1371/journal.pntd.0001207 (PMC3119642; doi:10.1371/journal.pntd.0001207)
Supplement: List S2 — 27 documents used in partial quantitative assessments but not submitted to full data extraction. (DOC) [file pntd.0001207.s004.doc]

**Supporting Information**

**List S2: 27 documents used in partial quantitative assessments but not submitted to full data extraction**

Almeida CE, Vinhaes MC, Almeida JR, Silveira AC, Costa J (2000) Monitoring the domiciliary and peridomiciliary invasion process of *Triatoma rubrovaria* in the State of Rio Grande do Sul, Brazil. Mem Inst Oswaldo Cruz 95(6): 761–768.

Áñez N, Crisante G, Rojas A (2004) Update on Chagas disease in Venezuela – A review. Mem Inst Oswaldo Cruz 99(8): 781–787.

Áñez N, Crisante G, Rojas A, Diaz N, Áñez-Rojas N, et al. (2003) La cara oculta de la enfermedad de Chagas en Venezuela. Bol Dir Malariol Salud Ambient 43(2): 45–57.

Ávila Montes GA, Martínez Hernández M, Ponce C, Ponce E, Soto Hernández R (1998) La enfermedad de Chagas en la zona central de Honduras: conocimientos, creencias y prácticas. Rev Panam Salud Pública 3(3): 158–163.

Bos R (1998) The importance of peridomestic environmental management for the control of the vectors of Chagas’ disease. Rev Argent Microbiol 20(Suppl.1): 58–62.

Costa J, Almeida CE, Dotson EM, Lins A, Vinhaes M, et al. (2003) The epidemiologic importance of *Triatoma* *brasiliensis* as a Chagas disease vector in Brazil: a revision of domiciliary captures during 1993-1999. Mem Inst Oswaldo Cruz 98(4): 443–449.

Dias E, Dias JCP (1968) Variações mensais da incidência das formas evolutivas do *Triatoma infestans* e do *Panstrongylus megistus* no município de Bambuí, estado de Minas Gerais (II^a^ nota: 1951 a 1964). Mem Inst Oswaldo Cruz 66(2): 209–26.

Dias E, Pellegrino J (1948) Alguns ensaios com o “Gammexane” no combate aos transmissores da doença de Chagas. Bras Médico 62(18-20): 185–191.

Diotaiuti L, Azeredo BVM, Busek SCU, Fernandes AJ (1998) Controle do *Triatoma* *sordida* no peridomicílio rural do município de Porteirinha, Minas Gerais, Brasil. Rev Panam Salud Pública 3(1): 21–25.

García-Zapata MT, Marsden PD, Virgens D, Penna R, Soares VA, et al. (1986) O controle da transmissão da doença de Chagas em Mambaí-Goiás, Brasil (1982-1984). Rev Soc Bras Med Trop 19(4): 219–225.

García-Zapata MT, Schofield CJ, Marsden PD (1985) A simple method to detect the presence of live triatomine bugs in houses sprayed with residual insecticides. Trans R Soc Trop Med Hyg 79(4): 558–559.

Guillén G (2002) El control de La enfermedad de Chagas en Bolivia. In: Silveira AC, org. El control de la enfermedad de Chagas en los países del Cono Sur de América. Historia de una Iniciativa Internacional 1991/2001. Uberaba: OPS–UFTM. pp. 109–143.

Lorca M, Schenone H, Valdés Padilla J (2002) El control de la enfermedad de Chagas en Chile. In: Silveira AC, org. El control de la enfermedad de Chagas en los países del Cono Sur de América. Historia de una Iniciativa Internacional 1991/2001. Uberaba: OPS–UFTM. pp. 251–268.

Marsden PD, Penna R (1982) A ‘vigilance unit’ for households subject to triatomine control. Trans R Soc Trop Med Hyg 76(6): 790–792.

Marsden PD, Virgens D, Magalhães I, Tavares-Neto J, Ferreira R, et al. (1982) Ecologia doméstica do *Triatoma infestans* em Mambaí, Goiás, Brasil. Rev Inst Med Trop São Paulo 24(6): 364–373.

Neghme A, Schenone H (1960) Resumen de 20 años de investigación sobre la enfermedad de Chagas en Chile. Rev Méd Chile 88(2): 82–93.

Pedreira de Freitas JL (1950) Resultados da aplicação de Rhodiatox e Gamexane contra triatomídeos. Rev Paulista Med 36: 231–243.

Ponce C, Ponce E, Flores M, Ávila G (1993) Intervention trials of new tools to control transmission of Chagas disease in Honduras. Mem Inst Oswaldo Cruz 88(Suppl.): 57–8.

Reithinger R, Ceballos R, Stariolo R, Davies CR, Gürtler RE (2006) Extinction of experimental *Triatoma infestans* populations following continuous exposure to dogs wearing deltamethrin-treated collars. Am J Trop Med Hyg 74(5): 766–771.

Rojas de Arias A (2001) Chagas disease prevention through improved housing using an ecosystem approach to health. Cad Saúde Pública 17(Suppl.): S89–S97.

Rojas de Arias A, Russomando G (2002) El control de la enfermedad de Chagas en Paraguay. In: Silveira AC, org. El control de la enfermedad de Chagas en los países del Cono Sur de América. Historia de una Iniciativa Internacional 1991/2001. Uberaba: OPS–UFTM. pp. 269–300.

Romaña C, Ábalos JW (1948) Acción del “Gamexane” sobre los triatomídeos. Control domiciliario. An Inst Med Reg Tucumán 2(2): 95–106.

Salvatella R (2002) El control de la enfermedad de Chagas en Uruguay. In: Silveira AC, org. El control de la enfermedad de Chagas en los países del Cono Sur de América. Historia de una Iniciativa Internacional 1991/2001. Uberaba: OPS–UFTM. pp. 301–316.

Segura EL (2002) El control de la enfermedad de Chagas en la República Argentina. In: Silveira AC, org. El control de la enfermedad de Chagas en los países del Cono Sur de América. Historia de una Iniciativa Internacional 1991/2001. Uberaba: OPS–UFTM. pp. 45–107.

Souza AG, Valério-Wanderley DM, Buralli GM, Andrade JCR (1984) Consolidation of the control of Chagas’ disease vectors in the state of São Paulo. Mem Inst Oswaldo Cruz 79(Suppl.): 125–131.

Wanderley DMV, Silva AR, Barbosa GL, Rodrigues VLCC, de Carvalho ME (2009) Doença de Chagas no estado de São Paulo: dos primórdios do controle vetorial à vigilância sustentável. Cad Saúde Colet 17(4): 857–872.

Wisnivesky-Colli C, Paulone I, Chuit R, Pérez A, Segura EL (1988) A new method for the detection of reinfested households during surveillance activities of control programmes of Chagas’ disease. Rev Argent Microbiol 20(Suppl.1): 96–102.
